# Effects of Increasing Doses of Lactobacillus Pre-Fermented Rapeseed Product with or without Inclusion of Macroalgae Product on Weaner Piglet Performance and Intestinal Development

**DOI:** 10.3390/ani10040559

**Published:** 2020-03-27

**Authors:** Gizaw Dabessa Satessa, Paulina Tamez-Hidalgo, Søren Kjærulff, Einar Vargas-Bello-Pérez, Rajan Dhakal, Mette Olaf Nielsen

**Affiliations:** 1 Department of Veterinary and Animal Sciences, University of Copenhagen, Grønnegårdsvej 3, DK-1870 Frederiksberg C, Denmark; evargasb@sund.ku.dk (E.V.-B.-P.); dhakal@sund.ku.dk (R.D.); 2 Fermentationexperts A/S, Vorbassevej 12, DK-6622 Copenhagen, Denmarkskj@fermbiotics.com (S.K.); 3 Department of Animal Sciences, Faculty of Technical Sciences, Aarhus University, Blichers Allé 20, 8830 Tjele, Denmark

**Keywords:** fermented feed, *Ascophyllum nodossum*, villi development, gut health

## Abstract

**Simple Summary:**

This study investigated the effects of dietary addition of increasing amounts (8, 10, 12, 15 and 25%) of pre-fermented rapeseed meal (FRM) or 10% FRM with the inclusion of 0.6% or 1.0% *Ascophyllum nodosum* (AN), a brown macroalgae, on performance and gut health as compared to either a non-supplemented diet (negative control, NC) or a diet supplemented with 2500 ppm ZnO (PC) in piglets weaned at 28 days of age. At any of the amounts supplemented, FRM sustained growth performance similar to the PC group during the first 10 days before weaning (18–27 days of age) and improved performance better than the PC from 28–41 days of age when fed at 8%. Inclusion of AN (0.6% or 1.0%) on top of 10% FRM did not affect growth performance. The percent of piglets that completed the experiment was increased at all levels of FRM (maximum of 91% at 8% FRM) or a combination of 10% FRM with AN (maximum of 90% at 10% FRM + 0.6% AN). Maximum intestinal development (villus, crypts, enterocytes) was observed at lower levels (8–10%) of FRM supplementation, but this was abolished by inclusion of AN. Feeding of FRM with or without AN increased some hematological parameters at all doses and enhanced immunoglobulin and interleukin-6 titers at lower doses (8% or 10%). In conclusion, FRM sustained piglet growth performance and intestinal development similar to ZnO with an optimal dietary inclusion level at 8–10% of dietary DM.

**Abstract:**

This study evaluated the effects of increasing doses of pre-fermented rapeseed meal (FRM) without or with inclusion of the brown macroalgae *Ascophyllum nodosum* (AN) on weaner piglets’ performance and gut development. Ten days pre-weaning, standardized litters were randomly assigned to one of nine isoenergetic and isoproteic diets comprising (on DM basis): no supplement (negative control, NC), 2500 ppm ZnO (positive control, PC), 8, 10, 12, 15 or 25% FRM, and 10% FRM plus 0.6 or 1.0% AN. Fifty piglets receiving the same pre-weaning diets were weaned at 28 days of age and transferred to one pen, where they continued on the pre-weaning diet until day 92. At 41 days, six piglets per treatment were sacrificed for blood and intestinal samplings. The average daily gain was at least sustained at any dose of FRM (increased at 8% FRM, 28–41 days) from 18–41 days similar to PC but unaffected by inclusion of AN. The percentage of piglets that completed the experiment was increased by FRM compared to NC, despite detection of diarrhea symptoms. FRM showed quadratic dose-response effects on colon and mid-jejunum crypts depth, and enterocyte and mid-jejunum villus heights with optimum development at 8% or 10% FRM, respectively, but this was abolished when AN was also added. In conclusion, FRM sustained piglet growth performance and intestinal development similar to ZnO with an optimum inclusion level of 8–10% of dietary DM.

## 1. Introduction

Weaning is the most challenging event in modern pig production, which often results in reduced feed intake, decreased growth, increased risks of post-weaning diarrhea (PWD) and mortality in newly weaned piglets [1,2,3]. Thus, pig producers in Europe, particularly in Denmark, heavily depend on dietary supplementation of pharmacological doses (2500 to 3000 ppm) of zinc oxide (ZnO) to avert weaning related diarrhea and associated piglet mortality. However, due to environmental concerns [4] and increased risk of antibiotic-resistant bacteria [5,6], the European Union passed a new law in July 2017, which bans the use of in-feed ZnO at pharmacological doses beyond 2022 [7]. As a consequence, pig producers will face huge challenges to sustain piglet performance and prevent PWD and associated mortality unless effective alternative feeding strategies are developed. 

Dietary inclusion of feeds pre-fermented with probiotic bacteria could be a promising dietary strategy to replace in-feed ZnO in weaner piglet diets. A meta-analysis-based investigation revealed that fermented feeds increased growth performance irrespective of age when fed to pigs by reducing intestinal coliforms, increasing villi development, and hence nutrient absorption, and enhancing immune responses [8]. Gut health has also been improved during weaning when pre-fermented diets have been fed to newly weaned piglets due to increased abundance of beneficial bacteria, such as lactic acid bacteria, and decreased numbers of enteropathogenic bacteria such as *Eschdurierichia coli* and overall coliforms throughout the gastrointestinal tract [9,10,11]. Unlike non-fermented products, fermented feed has been shown to lower fecal excretion of pathogenic coliforms when fed to sows [12]. 

Recently, rapeseed, particularly in its fermented form, has attracted interest in monogastric animals such as poultry and pigs as an alternative protein source as well as for manipulation of gut health. Feeding *Lactobacillus* pre-fermented rapeseed meals (FRM) to broiler chicken has been shown to increase feed digestibility and utilization (by reducing impact of anti-nutritional factors), gut morphology and antioxidant capacity in the body [13]. We have also previously tested FRM or rapeseed meal co-fermented with the brown macroalgae *Ascophyllum nodosum* (AN) and or *Saccharina latissima* (SL) in weaned piglets where 10% FRM inclusion improved growth performance, intestinal health and colon microbiota. However, the inclusion of either AN or AN plus SL in FRM resulted in inconsistent and contradictory findings between growth performance and gut health indices [14]. 

Nevertheless, brown macroalgae are currently viewed as another promising dietary strategy to improve growth performance and gut health in weaned piglets due to their wide range of bioactive components [15]. The bioactive carbohydrate components from brown macroalgae, such as laminarin and fucoidan, are unique in that they are not found in terrestrial plants and possess antimicrobial [16,17], immunomodulatory [18] and antioxidant [19] properties. Inclusion of these purified extracts in weaner piglets’ diets also improved overall growth performance [20,21], but are relatively expensive compared to the whole plant due to the cost of extraction. Available studies involving intact macroalgae, which are limited in contrast to purified extracts, have reported contradicting findings on the growth performance and gut health in pigs [22,23,24]. These variations could be attributed to, among others, the amount and species of macroalgae administered. In our previous study, we observed that the positive effects of FRM were constrained following inclusion of either AN or AN plus SL. However, the optimum doses for both FRM and the macroalgae products were generally unknown. 

Thus, we hypothesized that: (1) At optimal dietary inclusion levels, FRM can sustain weaner piglet performance, promote gut development and prevent PWD as efficiently as dietary addition of pharmacological doses of ZnO (2500 ppm), (2) inclusion of the brown macroalgae AN can act synergistically with FRM at a lower but optimal dose to promote weaner piglet performance and health.

Therefore, the objectives of this pilot study were (1) to investigate the effects of increasing doses of FRM on weaner piglet performance and intestinal development as compared to medicinal ZnO or no supplement, and (2) to investigate the potential synergistic effects between AN and FRM when AN was included at lower doses. 

## 2. Materials and Methods

This study was carried out at a commercial pig farm (Chotycze Farm, Łosice, Poland) from September 2017 to March 2018. The experiment was approved by the local ethics committee for animal experiments of the University of Lublin with authorization number 67/2017.

### 2.1. Experimental Design, Animals and Feeding 

A total of 450 piglets (Landrace & Yorkshire hybrid [LY] × Duroc & Pietrain hybrid [DP] crossbreed) were recruited for the study from 45 sows (LY Danbred C22 line, DanBred, Denmark) in their second or third parity. The piglets came from litters that were standardized to 14 piglets per sow. Standardization of litters to 14 piglets per sow was made by transferring piglets among sows until 14 days of age. Thereafter, no movement of piglets among sows was allowed. At 18 days of age (10 days before weaning), piglets were introduced to one of nine experimental diets comprising a basal diet (pre-starter, 18 to 64 days of age or starter diet, 65 to 92 days age) with: no supplement (NC), 2500 ppm (PC), increasing levels of FRM (8, 10, 12, 15 or 25% of dietary DM), and a combination of 10% FRM with either 0.6 or 1.0% AN of dietary DM while still with the sows, with piglets from five sows receiving the same experimental diet. At 28 days of age, the piglets were weaned and 50 of the piglets already receiving the same dietary treatment, excluding the runts, were then transferred from five sows to one slatted floor pen in the weaner unit per treatment and continued on the same dietary treatment until they exited the weaner unit at 92 days of age. All diets were based on the same basal diet, where wheat and soybean products were replaced with increasing amounts of FRM and AN. 

All sows received the same standard lactation diet commonly used at the farm. Piglets were fed pre-starter diets from 18 (10 days pre-weaning) to 64 days of age, and a starter diet from 65 to 91 days of age, i.e., until the end of the experiment, ad libitum from open troughs along with ad libitum access to water from drinking nipples during the entire trial.

### 2.2. Preparation of Experimental Diets 

FRM (commercial name EP100i) was obtained from European Protein (Baekke, Denmark). It was prepared with solid-state fermentation using inocula from three lactic-acid fermentative bacteria, namely, *Pediococcus acidilactici* (DSM 16243), *Pediococcus pentosaceus* (DSM 12834) and *Lactobacillus plantarum* (DSM 12837) where ground rapeseed meal (80% on DM basis) was mixed with the inoculant broth in a one-step fermentation process with constant stirring, followed by decanting and incubation at 38 °C for four days. 

The mixing of the final experimental diets was conducted by a certified feed company where no heat treatment was involved after the addition of *Lactobacillus* spp. containing FRM product. Formulation of the diets was performed by AgroSoft® (Tørring, Denmark) according to the Danish nutritional recommendations for piglets [25]. Analyses for energy contents and nutritional composition of the final pre-starter and starter diets were conducted at the commercial lab Dolfos® (Piotrków Trybunalski, Poland). Both the pre-starter and starter diets were designed to be iso-proteic and iso-energetic across treatments despite the presence of some deviation in the final products as shown in Table S1.

### 2.3. Recordings of Feed Intake and Body Weight

Piglets were ear-tagged at birth, and body weights of individual piglets were recorded 10 days before weaning (18 days of age), at weaning (28 days of age, and 14 days post-weaning (41 days of age). Thereafter, body weight was measured in each treatment on pen-basis until the piglets exited the experiment at 92 days of age. Feed consumption in each experiment was recorded daily on pen-basis throughout the experiment.

### 2.4. Piglets’ Health Assessment and Fecal Sampling 

Piglets were examined daily by farm staff for signs of diarrhea or signs of any other ailments including mortality and if possible, its causes, and any changes observed were recorded. Piglets with poor health conditions due to, among others, diarrhea were removed from the pen and experiment to be treated with antibiotics elsewhere. No antibiotics treatment was allowed for any piglet remaining in the experiment. Fecal samples were collected from each pen and analyzed for lactic acid content at the veterinary diagnostic laboratory, ALAB Weterynaria (Warsaw, Poland). A completion rate was calculated in percentage for each treatment based on the number of piglets remaining in a pen at the end of the experiment and the number of piglets assigned to pens at weaning. 

### 2.5. Blood and Intestinal Samplings from Subgroups of Piglets

Fourteen days after weaning (41 days of age), six piglets were randomly selected from each treatment or pen, and transported to a commercial slaughter facility. Piglets were sacrificed by exsanguination following captive bolt stunning. Whole blood samples were collected during exsanguination from the jugular vein into ethylene diamine tetra acetic acid (EDTA) tubes for hematological analysis and plain vacutainer tubes for analysis of serum immunoglobulins and interleukin-6. Whole blood samples from EDTA tube were stored at +4 °C and the blood samples in plain tubes were allowed to clot to separate the serum and the serum samples were then stored at –20 °C until analyses the following day. 

The abdominal wall was opened, and the cardiac sphincter, pyloroduodenal, ileocecal and anorectal junctions were ligated, and the gut segments were cut and taken out. Tissue sections of approximately 2 cm length were sampled from the middle of the small intestine and the apex of the ascending colon. Sampled tissue sections were gently rinsed for intestinal contents with saline and fixed in 10% buffered formalin. 

### 2.6. Intestinal Morphology, Haematology and Serum Immunoglobulins

Histological analyses of mid-jejunum and colonic tissue were conducted at the commercial laboratory ALAB Weterynaria (Warsaw, Poland) using a standard light microscope Olympus BX41 and CellSens software (Olympus Corporation, Tokyo, Japan). All microscopic evaluations were performed in a blinded fashion. Initially, tissue samples fixed in 10% buffered formalin were dehydrated using graded ethanol and xylene baths and embedded in paraffin wax. Tissue sections of 3–4 µm were mounted on the microscopic slide and stained with hematoxylin and eosin (HE). General histopathological examinations were evaluated at a magnification of 10×, 40× and 100× (objective lens) and 10× (eyepiece), and photographic documentation was made. For the mid-jejunum the following were examined: the mucosa and submucosa, intestinal crypts, mucosal and submucosal blood vessels, enterocyte height and brush border integrity as well as the presence of goblet cells. The morphometric analysis delivered the following quantitative results: mid-jejunum villus height (JVH) at 10× magnification, mid-jejunum crypt depth (JCD) and colonic crypt depth (CCD) both at 10× magnification, and mid-jejunum enterocyte height (JEH) at 40× magnification. Two types of scoring analyses were performed: for intraepithelial lymphocytes infiltration (IEL) and stromal lymphocytes infiltration (SL). The following scale was used in both scoring evaluations: 0—normal; 1—slight; 2—moderate; 3—severe. 

Hematological analysis of whole blood samples and serum immunoglobulin analysis were conducted at the ALAB Weterynaria (Warsaw, Poland). Analysis of red blood cell counts (RBC), hematocrit value (HCT), hemoglobin content (Hb), white blood cell counts (WBC) and differential leucocytes count were carried out using a Sysmex XT 2000i analyzer (Sysmex Corporation, Kobe, Japan). Commercially available swine-specific enzyme-linked immunosorbent assay (ELISA) kits (Cusabio®, Houston, TX, USA) were employed to quantify the concentrations of serum immunoglobulins such as immunoglobulin G (IgG, Catalog Number: CSB-E06804p), immunoglobulin A (IgA, Catalog Number: CSB-E13234p) and immunoglobulin M (IgM, Catalog Number: CSB-E06805p) as well as the pro-inflammatory cytokine interleukin-6 (IL-6, Catalog Number: CSB-E06786p) according to the manufacturer protocols.

### 2.7. Data Analyses

Data for growth performance, hematology and serum immunoglobulins, as well as a dose response effect of FRM on gut morphometry were analyzed using Linear Mixed Models of R version 3.6.0 [26]. Dietary treatment effects were evaluated using the following statistical model, and differences between individual treatments were assessed by using Tukey’s Honest Significance Difference Test:

(1) $$Yijkl=µ+T_{i}+S_{j}+{\left(TxS\right)}_{ij}+{\left(W+W^{2}\right)}_{k}+P_{l}+\varepsilon_{ijkl}$$

where, Yijkl is the response variable, µ is the overall mean, $T_{i}$ is the effect of dietary treatment (i = 1, 2, 3, …, 9), $S_{j}\;$ is the effect of sex of piglets (*j* = male or female), ${\left(TxS\right)}_{ij}$ is the treatment and sex interaction, ${\left(W+W^{2}\right)}_{k}\;$ is the linear and quadratic effects, respectively, of body weight at the start of the experiment (18 days post-partum) (*k* = 1,2,3,…, 50), $P_{l}$ is the random effects of individual piglets and $\varepsilon_{ijkl}$ is the residual.

Linear and quadratic relations for dietary inclusion level of FRM (FRM dose as % of dietary DM) and effects on gut morphometric parameters were analyzed using the following model:

(2) $$Yijklm=µ+T_{i}+S_{j}+{\left(TxS\right)}_{ij}+{\left(W+W^{2}\right)}_{k}+{\left(D^{2}\right)}_{l}+P_{m}+\varepsilon_{ijklm}$$

where, Yijklm is the response variable, µ is the overall mean, $T_{i}$ is the effect of dietary treatments (I = 1, 2, 3, …., 9), $S_{j}$ is the effect of sex of the piglet (j = male or female), ${\left(TxS\right)}_{ij}$ is the treatment and sex interaction, ${\left(W+W^{2}\right)}_{k}\;$ is the linear or quadratic effects, respectively, of body weight at the start of the experiment (18 days post-partum) (k = 1,2,3,…, 50), ${\left(D^{2}\right)}_{l}$ is the quadratic effects of the doses of the treatments, $P_{m}$ is the random effect of individual piglets and $\varepsilon_{ijklm}$ is the residual. The normality of the data was tested using normal quantile-quantile (Q-Q) plots. All non-normal data were log or box-cox (only in case of gut morphometry) transformed before statistical analysis. The best-fitting model was recruited based on Akaike information criterion (AIC) value. All results were expressed as least-square means (LSM) and standard error of the mean (SEM), and significance was considered at *p* < 0.05.

## 3. Results

### 3.1. Performance of Piglets 

As shown in Table 1, piglets supplemented with ZnO (PC) showed no difference in average daily gain (ADG) and BW except for a significant increment (*p* < 0.05) during the 10 days before weaning (18 to 27 days of age) and at 27 days of age, respectively, compared to the NC piglets. FRM sustained similar ADG during 18–27 and 18–41 days of age, and BW at 27 and 41 days of age as the PC at all doses, and significantly increased (*p* < 0.05) ADG during 28–41 days of age when supplemented at 8%. ADG was increased during 18–27 days of age, and demonstrated no discernable pattern (despite significant increment at 8% FRM) during 28–41 days of age, and showed no difference during 18–41 days of age with increasing doses of FRM. Inclusion of AN on top of 10% FRM at either dose did not affect ADG compared to the 10% FRM. Results for BW and ADG after 41 days of age (42–92 days of age) were pen-based single observation per treatment, and hence, could only provide an inconclusive rough estimation of growth (Table S2). Based on this estimation, FRM resulted in numerically higher ADG at all doses compared to the controls (NC and PC) during 42–64 days of age and similar ADG to PC during 65–92 days of age when fed at 10% dietary DM. FRM resulted in the highest ADG during both periods (42–64 and 65–92 days of age) when supplemented at 10% dietary DM. In this study, average daily feed intake and feed conversion ratio were not provided, as daily feed intake was measured on pen-basis only (single observation per treatment), and hence, it was impossible to quantify the exact amount of feed consumed by each piglet housed in a group.

### 3.2. Incidence of Diarrhea and Fecal Lactic Acid Concentration

Due to the limited number of observations per treatment, statistical analyses were not applied to data from diarrhea and fecal lactic acid concentrations. The 2500 ppm ZnO (PC) resulted in a numerically lower incidence and higher completion rates than NC. At any of the doses supplemented, FRM without or with AN resulted in a higher completion rate than the NC but achieved similar completion rates to that of PC (Table S3). Dietary lactic acid concentration was increased with increasing doses of FRM (despite some fluctuation at 15% supplementation) demonstrating 3–10 and 4–12 fold increases compared to the NC and PC, respectively. It also showed 5–6 fold and 6–7 folds increment compared to the NC and PC, respectively, with inclusion of AN on top of 10% FRM (Figure S1A). Supplementation with FRM also resulted in lactic acid concentrations in the feces of a 10–20-fold increase compared to the NC and a 5–10-fold increase compared to the PC. The fecal lactic acid concentration was markedly increased with an increasing dose of FRM (except for 15% FRM) but was reduced when AN was added (Figure S1B).

### 3.3. Gut Tissue Morphometry and Immune Cell Infiltration 

**Figure 1:**
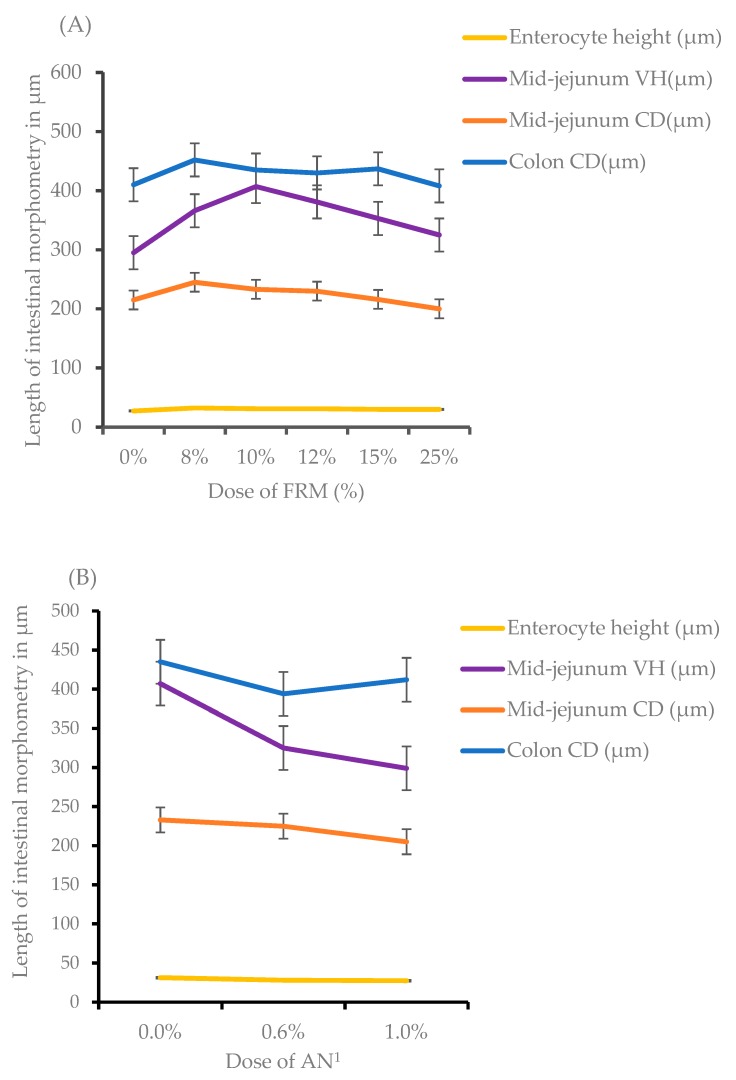
Effect of increasing doses of prefermented rapeseed meal (FRM) (**A**) or combination of 10% FRM with increasing doses of *Ascophyllum nodosum* (AN) (**B**) on enterocyte height, colon crypt depth, mid-jejunum crypt depth and villi in weaned piglets sacrificed at 14 days post-weaning. CD = crypt depth; VH = villus height; ^1^ AN was included on top of 10% FRM at 0.0%, 0.6% or 1.0% giving 10% FRM +0.6% AN or 10% FRM + 1.0% AN overall supplements.

**Figure 2:**
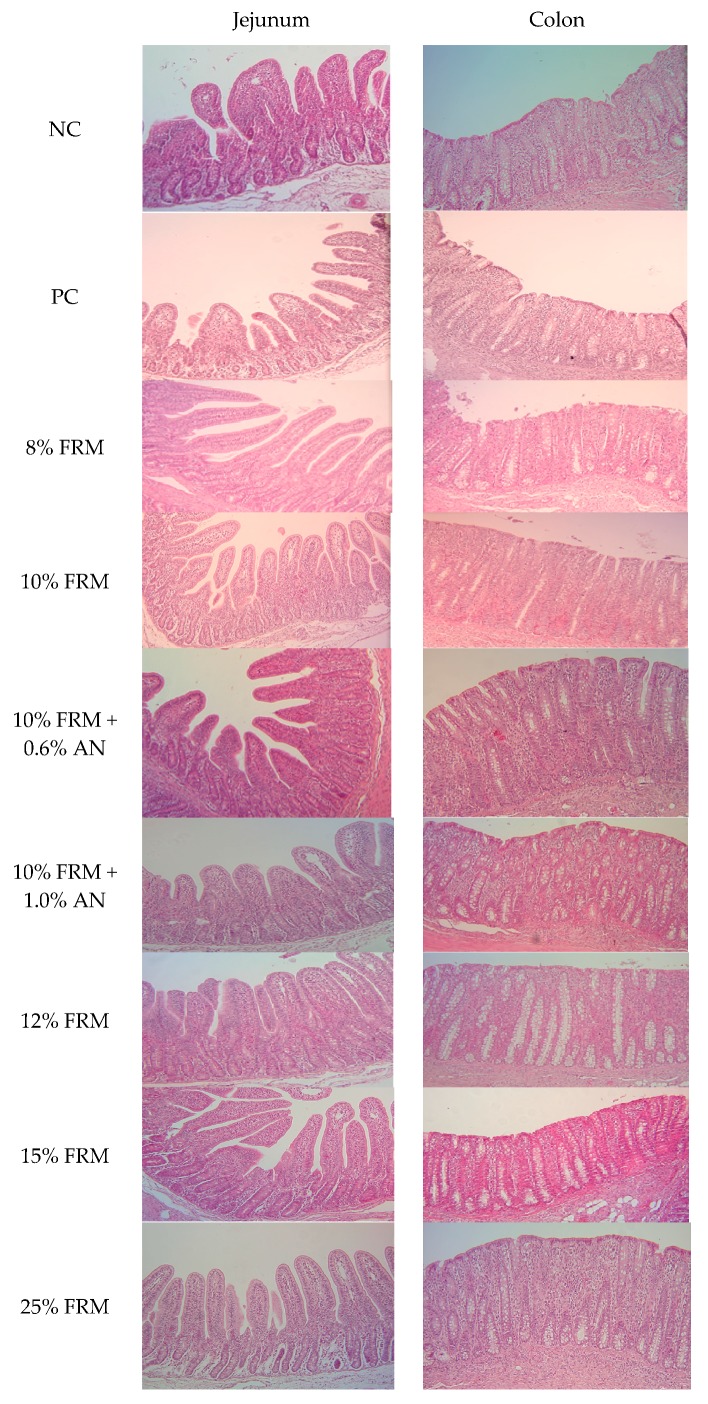
Effects of a pharmacological dose of ZnO, increasing doses of pre-fermented rapeseed meal (FRM) or combination of 10% FRM with increasing doses of *Ascophyllum nodosum* (AN) on the histomorphological features in the jejunal and colonic tissues in piglets 14 days after weaning. NC = negative control (basal diet without additives); PC = positive control (basal diet supplemented with 2500 ppm ZnO). Histomicrographs were taken at 10× magnification.

The effects of dietary supplementation on intestinal morphologies are shown in Figure 1, and the morphological features of intestinal tissues obtained from different dietary treatments are illustrated in Figure 2. Increasing doses of FRM resulted in quadratic effects on jejunum enterocyte height, villus height and crypt depth as well as colon crypt depth with maximum effects being observed at 8% FRM for colon and jejunal crypt depths, and 10% FRM for jejunal enterocyte and villus heights (Figure 1A) in weaned piglets 14 days after weaning. Inclusion of increasing doses of AN in 10% FRM counteracted all of these changes in intestinal histomorphometric features (Figure 1B). These intestinal histomorphometric features were not affected by the supplementation of ZnO compared to the NC. 

Supplementation of FRM at any dose or its 10% combination with any dose of AN resulted in numerically lower gut-associated lymphoid tissue (GALT) counts, and reduced immune cell infiltrations of the jejunal and colonic mucosa compared to the NC and PC piglets, which had similar levels of infiltration (Figure S2).

Assessment of histopathological changes revealed weak to strong stromal and intraepithelial immune cell infiltrations of the small intestine in both NC and PC piglets. A few piglets demonstrated disruption of the epithelial barrier and moderately visible brush borders in areas of significant intraepithelial lymphocyte infiltration in both controls. There were negligible immune cell infiltrations of the small intestine and colon at any doses of FRM compared to NC and PC, and infiltrations were limited only to the stroma in both intestinal segments. Inclusion of AN did not have significant impacts except for a slight increment in colonic stromal immune cell infiltration.

### 3.4. Hematological and Immunological Parameters

As shown in Table 2, FRM increased erythrocyte indices, WBC and lymphocyte (LYM) counts (*p* < 0.05), but reduced granulocyte (GRA) counts (except for 25% FRM) (*p* < 0.05) at any dose administered compared to NC and PC piglets. Inclusion of AN had no additional impact on hematological parameters except for a significant increment of RBC counts when 1.0% AN was supplemented. There were no differences in hematological values between PC and NC piglets except for higher WBC in NC (*p* < 0.05). 

As shown in Table 3, PC piglets had higher plasma IgG, IgA and IgM titers, but not IL-6 level, compared to NC piglets. The highest immunoglobulins and IL-6 titers were reached when weaner diets contained 8% or 10% FRM. Inclusion of AN significantly enhanced IgG titers but significantly reduced that of IL-6 compared to 10% FRM. Inclusion of AN on top of 10% FRM increased IgG level but decreased blood levels of IgA, IgM and IL-6.

## 4. Discussion

In the current study, FRM demonstrated the same desirable effects on ADG, and intestinal development and barrier function as we have previously observed and optimum effects were observed when 8% or 10% FRM was included in the weaner diets [14]. Similar improvements in ADG upon dietary addition of FRM in broiler chicken [27] and fermented soybean meal in piglets [28,29] were reported in other studies. This beneficial effect could most likely be explained by the fact that supplementation of fermented feed may provide beneficial probiotic bacteria, such as lactic acid bacteria, and also prebiotic products produced during the pre-fermentation process [30,31]. Rapeseed meal has a relatively high fiber and anti-nutritional contents compared to soybean, which can potentially decrease growth performance in monogastric species [32]. However, the negative effects of these components may be removed by pre-fermentation of rapeseed meal so that nutrient digestibility of the product could be increased [33], while anti-nutritional factors such as tannin, phytate and glucosinolate could be degraded during the pre-fermentation process [13,31]. 

In this study, AN did not have any impacts on ADG at any of the doses provided on top of the FRM. This is in contrast to Turner et al. [34], who reported a quadratic effect on ADG in piglets with increasing dietary levels of AN, with a maximum effect being achieved at 1% AN in dietary DM. However, Michiels et al. [24] have also found that feeding of intact AN to weaned piglets did not affect ADG and FCR, and supplementation of increased doses of AN to grower-finisher pigs has been reported to have a directly negative effect on ADG without affecting FCR and feed intake [22]. The lack of effects of intact seaweeds on piglet performance in the current study may be due to the presence of a large amount of anti-nutritional factors, such as phlorotannins in the intact macroalgae [35], and potentially also due to antagonistic actions of bioactive components in the algae. In some studies, purified extracts of special carbohydrates from macroalgae, such as laminarin and fucoidan, have been shown to improve ADG and overall performance of the animal when fed alone, but not when fed in combination [20,36]. Furthermore, the antimicrobial activities of AN, as confirmed by a drastic reduction in the concentration of fecal lactic acids compared to FRM, NC and PC piglets, may reduce hindgut fermentation and thus the availability of SCFAs and microbial vitamins that are essential for growth and also intestinal health (Figure S1B). 

Inclusion of FRM at 8% or 10% of dietary DM in weaner diets in the current study resulted in improved intestinal morphometric indices (JVH, JEH, JCD and CCD), intestinal mucosal integrity and barrier functions consistent with observed improvements in ADG. Long villus height and intact mucosal integrity are essential for nutrient digestion and absorption as well as prevention of invasion of intestinal tissues by pathogenic microbes. Similar findings were reported when fermented rapeseed was fed to broilers where intestinal villus height and villus-to-crypt ratio showed significant improvements [13,37,38]. Feeding of fermented soybean to weaned piglets [39] and Japanese quail [40] also demonstrated significant improvement in villus height but reduced crypt depth along the intestine. However, some studies showed that feeding of lactobacilli fermented liquid feed (feed to water ratio of 1:3) significantly reduced JVH in weaned piglets [41]. The increase in villi development could be due to reduced anti-nutrition factors by pre-fermentation of the feed product [13,42] or increased fermentation products, mostly SCFAs, such as lactate, acetate and butyrate [43]. Stresses during weaning can increase oxidative stresses, which may lead to damage of intestinal tissues during weaning [44], but the generation of metabolites with antioxidant potential during fermentation could also be responsible for the healthy mucosal integrity and barrier functions observed in the current study [45].

The desirable effects of FRM on gut histomorphometry and GALT were counteracted by the addition of AN on top of 10% FRM. A similar result was reported where intestinal villus height was reduced in response to feeding of weaned piglets with a seaweed extract from *Laminaria digitata* [21]. In contrast, some studies reported villus height and villus-to-crypt ratio were improved when weaned piglets were supplemented with the seaweed extracts such as laminarin and fucoidan separately but these morphometric parameters were not affected when these extracts were supplemented in combination [46,47].

Although a high percentage of piglets completed the experiment in response to feeding of FRM (highest at 8% FRM) or a combination with AN (highest at 0.6% AN), piglets were not protected from diarrhea in the current study. We speculate that this diarrhea could be linked to osmotic pressure triggered by possibly high intestinal lactic acid concentrations as confirmed by detection of high fecal lactic acid concentration amidst intact intestinal mucosal integrity and barrier functions. In contrast, fermented feed such as soybean has been reported to prevent diarrhea in piglets weaned at 28 days of age [28,48]. Studies also revealed that the inclusion of fermented feeds in the diets of monogastric animals reduced the counts of enteric pathogens such as *Salmonella typhimurium* and *E. coli*, which are known etiologic agents for enteric infections and diarrhea [28,49,50]. Regarding the macroalgae AN, studies are limited and available ones reported inconsistent findings, where feeding of AN to growing–finisher pigs reduced coliform counts [22], while no effect on gut microbial profile was reported when AN was fed to weaned piglets [24]. However, others found that feeding of purified seaweeds extracts such as laminarin and fucoidan reduced enteropathogens such as *E. coli* which are the causative agent of infectious diarrhea during weaning [18,51]. 

RBC indices are essential markers for efficient oxygen transport to tissues and nutrient utilization for energy release. Although all of the RBC indices investigated were in the normal ranges for all of the treatments in the current study [52], they were increased in piglets fed FRM without or with AN compared to the NC and PC. Bhattarai and Nielsen [53] reported that a positive association exists between growth and hematological indices following weaning. Thus, it could be speculated that the increase in RBC indices demonstrated in the present study might be associated with increased growth and hence metabolic demands in pigs fed with FRM or its combination with AN. 

In the current study, we found out that WBC indices were generally within normal ranges compared to reference values [52], and feeding of FRM and PC reduced WBC counts in weaned piglets in contrast to the NC. Conversely, levels of serum immunoglobulins (IgG, IgM, IgA) and IL-6 were increased in response to lower doses (8% or 10% FRM), while the inclusion of AN enhanced IgG but reduced the rests. A study showed that feeding of fermented products, such as the soybean meal, to weaned piglets significantly improved mucosal or systemic immunoglobulins [38,39,54,55]. The discrepancy between increased immunoglobulins and WBC counts could be related to the preferential activation of the adaptive immune responses, which culminate in the increment of immunoglobulins. Serum immunoglobulins may also increase in response to probiotic bacterial such as lactic acid bacteria and also bioactive components present in FRM [31,38]. The weaning period is usually marked by an ineffective immune response due to the immaturity of the immune system and also depression by weaning stresses. However, appropriate immune responses during this period could be very essential to prevent infections by enteric pathogens, and hence post-weaning diarrhea.

## 5. Conclusions

FRM improved piglet performance (ADG) and intestinal histomorphometric indices demonstrating optimum effects at 8% or 10% inclusion, but further inclusion of the brown macroalgae *Ascophyllum nodosum* (AN) did not show any additional benefits. Thus, FRM could be a potential alternative for medicinal ZnO when supplemented at ≤ 10% dietary DM. However, a comprehensive dose-response study is deemed necessary to assure optimum inclusion rate for desirable effects on performance and gut health. 

## Figures and Tables

**Table 1:** Effects of dietary addition of increasing doses of *Lactobacillus* pre-fermented rapeseed meal (FRM) or a combination of 10% FRM with increasing doses of AN on growth performance in piglets weaned at 28 days of age.

Parameters	Treatments									SEM	*p*-Value	
	Controls		FRM (%)					AN ^1^ (%)				
	NC	PC	8	10	12	15	25	0.6	1.0		TG	IBW
BW at d 18, kg	5.83 ± 0.88	5.28 ± 0.78	5.62 ± 0.83	4.92 ± 0.87	4.92 ± 0.83	4.92 ± 0.91	5.22 ± 0.75	5.22 ± 0.99	5.21 ± 1.10			
Days 18–27												
BW at d 27, kg	6.67 ^a^	7.22 ^bcd^	6.99 ^ab^	6.95 ^ac^	7.22 ^bcd^	7.48 ^bd^	7.50 ^d^	7.29 ^cd^	7.36 ^bd^	0.092	***	***
ADG, g/d	143 ^a^	198 ^bcd^	175 ^ab^	181 ^bc^	198 ^bcd^	224 ^d^	226 ^d^	205 ^bcd^	212 ^cd^	8.1	***	***
Days 28–41												
BW at d 41, kg	7.78 ^a^	8.34 ^ab^	8.96 ^bc^	8.31 ^ab^	9.18 ^c^	8.79 ^bc^	9.01 ^bc^	8.91 ^bc^	8.96 ^bc^	0.19	***	***
ADG, g/d	108 ^ab^	100 ^a^	153 ^b^	106 ^ab^	148 ^ab^	99 ^a^	116 ^ab^	124 ^ab^	123 ^ab^	12.2	**	NS
Days 18–41												
ADG, g/d	110 ^a^	142 ^ab^	162 ^b^	141 ^ab^	164 ^b^	155 ^b^	164 ^b^	159 ^b^	162 ^b^	8.30	***	***

^a,b,c,d^ Different letters within same row indicate significant difference (*p* < 0.05); *** *p* ≤ 0.001; ** *p* ≤ 0.01; * *p* ≤ 0.05; NS = non-significant; ADG = average daily gain; IBW = initial body weight at 18 days of age; TG = treatment group; NC = negative control (basal diet without additives); PC = positive control (basal diet supplemented with 2500 ppm ZnO); AN = *Ascophyllum nodosum*; FRM = pre-fermented rapeseed meal (EP100i); ^1^ AN was included on top of 10% FRM at 0.6% or 1.0% giving 10% FRM +0.6% AN or 10% FRM + 1.0% AN overall supplements.

**Table 2:** Effects of a pharmacological dose of ZnO, increasing doses of *Lactobacillus* pre-fermented rapeseed meal (FRM) and combination of 10% FRM combination with increasing doses of *Ascophyllum nodosum* (AN) on hematological parameters in weaned piglets slaughtered 14 days post-weaning.

Parameters	Treatments									SEM
	Controls		FRM (%)					AN ^1^ (%)		
	NC	PC	8	10	12	15	25	0.6	1.0	
RBC indices										
RBC, 10^6^/µL	5.27 ^a^	5.46 ^a^	6.87 ^bc^	6.53 ^b^	6.87 ^bc^	6.68 ^b^	7.68 ^c^	6.88 ^bc^	7.67 ^c^	0.223
Hb, g/dL	8.48 ^a^	8.29 ^a^	11.12 ^b^	11.33 ^bc^	11.12 ^b^	11.85 ^bc^	12.37 ^c^	11.67 ^bc^	11.33 ^bc^	2.60
HCT, %	30.62 ^a^	32.83 ^a^	37.54 ^b^	37.76 ^b^	37.54 ^b^	39.65 ^b^	40.21 ^b^	37.81 ^b^	38.04 ^b^	0.891
Total differential WBC counts										
WBC, 10^3^/µL	34.54 ^d^	15.21 ^a^	20.9 ^bc^	19.60 ^ab^	18.60 ^ab^	22.20 ^bc^	26.00 ^c^	20.60 ^bc^	20.30 ^bc^	1.055
LYM, %	53.4 ^ab^	51.4 ^a^	62.7 ^bc^	62.9 ^bc^	69.5 ^c^	69.3 ^c^	62.5 ^bc^	62.9 ^bc^	64.8 ^c^	2.43
GRA, %	45.7 ^c^	45.0 ^bc^	34.7 ^a^	35.0 ^a^	27.1 ^a^	27.6 ^a^	35.5 ^ab^	34.5 ^a^	33.9 ^a^	2.451
MID, %	3.32	3.42	2.53	1.85	2.57	1.78	2.13	2.23	1.72	0.486

^a,b,c,d^ Different letters within same row indicate significant difference (*p* < 0.05); initial body weight (IBW) has significant linear effect on Hb and quadratic effect on HCT and LYM %; NC = negative control (basal diet without additives); PC = positive control (basal diet supplemented with 2500 ppm ZnO); IBW = initial body weight; WBC = white blood cell count; LYM = lymphocyte count; GRA = the number and percentage of granulocytes; MID = mid-range absolute count (indicate non-classified cells); RBC = red blood cells count; HCT = hematocrite; Hb = hemoglobin concentration; ^1^ AN was included on top of 10% FRM at 0.6% or 1.0% giving 10% FRM +0.6% AN or 10% FRM + 1.0% AN overall supplements.

**Table 3:** Effects of a pharmacological dose of ZnO, increasing doses of *Lactobacillus* pre-fermented rapeseed meal (FRM) and a combination of 10% FRM with increasing doses of *Ascophyllum nodosum* (AN) on immunological parameters in weaned piglets slaughtered 14 days post-weaning.

Parameters	Treatments									SEM	*p*-Value
	Controls		FRM (%)					AN ^1^ (%)			TG
	NC	PC	8	10	12	15	25	0.6	1.0		
IgG, mg/ml	2.11 ^ab^	2.39 ^cd^	3.63 ^f^	2.22 ^bc^	2.57 ^de^	1.94 ^a^	2.62 ^de^	2.57 ^d^	2.82 ^e^	0.062	<0.0001
IgA, mg/ml	0.099 ^bc^	0.136 ^d^	0.107 ^c^	0.170 ^e^	0.077 ^ab^	0.080 ^ab^	0.066 ^a^	0.137 ^d^	0.081 ^ab^	0.005	<0.0001
IgM, mg/ml	0.395 ^bc^	0.545 ^d^	0.427 ^c^	0.679 ^e^	0.309 ^ab^	0.318 ^ab^	0.263 ^a^	0.547 ^d^	0.323 ^ab^	0.022	<0.0001
IL-6, pg/ml	208.6 ^ab^	214.6 ^ab^	370.9 ^e^	260.0 ^c^	195.5 ^a^	226.8 ^b^	285.4 ^d^	224.8 ^b^	214.9 ^ab^	5.4	<0.0001

^a,b,c,d^ Different letters within same row indicate significant difference (*p* < 0.05); live body weight (LBW) at slaughter has no effect any of the immunological parameters; NC = negative control (basal diet without additives); PC = positive control (basal diet supplemented with 2500 ppm ZnO); LBW = live body weight; TG = Treatment group; ^1^ AN was included on top of 10% FRM at 0.6% or 1.0% giving 10% FRM +0.6% AN or 10% FRM + 1.0% AN overall supplements.

## Supplementary Materials

The following are available online at https://www.mdpi.com/2076-2615/10/4/559/s1, Figure S1: Effect pharmacological doses of ZnO, increasing doses of FRM or combination of 10% FRM with AN on dietary (Panel A) and fecal (PaneL B) lactic acid concentrations, Figure S2: Effects of various doses of FRM with or without AN on the density of GALT (Panel A), and jejunal (Panel B) and colonic (Panel C) immune cell infiltrations in piglets (LSM ± SEM) slaughtered two weeks post-weaning, Table S1: Ingredients and chemical compositions of the pe-starter and starter diets fed to piglets from 10 days before weaning until they exited the experiment at 92 days of age, Table S2: Effects of a pharmacological dose of ZnO, increasing doses of FRM and combination of 10% FRM with increasing doses of AN on growth performance in piglets weaned at 28 days of age, Table S3: Effects of a pharmacological dose of ZnO, increasing doses of FRM and a combination of 10% FRM with increasing doses of *Ascophyllum nodossum* on a number of cases of post-weaning diarrhea. The percentage of piglets that exited the weaner units are also shown.
