# Supplementary material for: Effects of Increasing Doses of Lactobacillus Pre-Fermented Rapeseed Product with or without Inclusion of Macroalgae Product on Weaner Piglet Performance and Intestinal Development
Source: Animals (Basel). 2020 Mar 27;10(4):559. doi: 10.3390/ani10040559 (PMC7222423; doi:10.3390/ani10040559)
Supplement: Supplementary file 1 [file animals-10-00559-s001.pdf]

Table S1. Ingredients and chemical compositions of the pe-starter and starter diets fed to piglets from 10 days before weaning until they exit the experiment at 92 days of age.

Name	Pre-starter diets									Starter diets								
	Controls		FRM (%)					AN¹ (%)		Controls		FRM (%)					AN¹ (%)	
	NC	PC	8	10	12	15	25	0.6	1.0	NC	PC	8	10	12	15	25	0.6	1.0
Barely	35.0	35.0	35.0	35.0	35.0	35.0	35.0	35.0	35.0	30.0	30.0	30.0	30.0	30.0	30.0	30.0	30.0	30.0
Wheat	23.7	22.3	28. 970	27.9	27.0	25.6	18.9	27.3	26.9	34.8	34.5	35.0	34.4	33.1	31.9	26.3	33.9	33.4
Soybean meal	13.0	13.1	3.9	2.7	1.5	0.9	0.0	2.6	2.6	15.4	15.4	12.3	10.7	9.9	8.0	3.7	10.6	10.6
Potato protein	2.50	2.50	2.50	2.50	2.50	2.50	2.50	2.50	2.50	2.50	2.50	-	-	-	-	-	-	-
Fishmeal	3.00	3.00	3.00	3.00	3.00	3.00	1.40	3.00	3.00	1.80	1.80	3.00	3.00	3.00	3.00	2.50	3.00	3.00
Whey powder	3.00	3.00	4.25	4.25	4.25	4.25	4.25	4.25	4.25	5.00	5.00	-	-	-	-	-	-	-
Whole milk powder	5.00	5.00	5.00	5.00	5.00	5.00	5.00	5.00	5.00			5.00	5.00	5.00	5.00	5.00	5.00	5.00
Vegetable oil	0.70	0.70	4.25	2.69	2.87	2.93	3.70	2.78	2.84	1.00	1.00	2.69	3.03	3.30	3.43	4.03	3.16	3.26
Lysine, L-Lys (98.5%)	0.74	0.73	0.76	0.76	0.76	0.76	0.80	0.75	0.75	0.60	0.60	0.62	0.62	0.61	0.63	0.64	0.63	0.62
DL-Met (99)	0.22	0.22	0.21	0.21	0.20	0.20	0.20	0.21	0.21	0.26	0.26	0.13	0.12	0.12	0.11	0.10	0.12	0.12
L-Thr (98.5%)	0.30	0.30	0.30	0.29	0.29	0.29	0.30	0.29	0.29	0.25	0.24	0.21	0.21	0.20	0.20	0.20	0.20	0.20
L-Trp (98%)	0.12	0.12	0.15	0.15	0.15	0.15	0.16	0.15	0.15	0.01	0.01	0.07	0.07	0.07	0.07	0.08	0.07	0.07
Calcium carbonate (36%)										0.45	0.45	0.4	0.33	0.33	0.3	0.24	0.31	0.28
Monocalcium phosphate	0.91	0.92	1.02	0.98	0.94	0.88	0.78	0.99	0.99	1.06	1.06	0.93	0.89	0.81	0.76	0.59	0.86	0.86
Sodium chloride	0.25	0.25	0.31	0.36	0.35	0.34	0.33	0.31	0.28	0.14	0.14	0.43	0.42	0.41	0.40	0.37	0.37	0.34
Calcium formate	0.40	0.40	0.70	0.70	0.70	0.70	0.70	0.70	0.70	0.20	0.20	0.50	0.50	0.50	0.50	0.50	0.50	0.50
Sodium bicarbonate	0.15	0.15								0.15	0.15	-	-	-	-	-	-	-
Iron fumarate 31%	0.25	0.25	0.25	0.25	0.25	0.25	0.25	0.25	0.25									
Pigor® dry aroma	0.20	0.20	0,200	0.20	0.20	0.20	0.20	0.20	0.20	-	-	0.20	0.20	0.20	0.20	0.20	0.20	0.20
EP premix	0.50	0.50	0.50	0.50	0.50	0.50	0.50	0.50	0.50	0.50	0.50	0.50	0.50	0.50	0.50	0.50	0.50	0.50
Sucram	0.07	0.07	0,070	0.07	0.07	0.07	0.07	0.07	0.07	-	-	-	-	-	-	-	-	-
EP200	-	-	-	2.50	2.50	1.50		2.50	2.50			-	-	-	-	-	-	-
Medicinal Zn		0.25								-	0.25	-	-	-	-	-	-	-
EP100			8.0	10.0	12.0	15.0	25.0	10.0	10.0	-	-	8.0	10.0	12.0	15.0	25.0	10.0	10.0
EP900								0.6	1	-	-	-	-	-	-	-	0.6	1
Calculation																		
Dry matter (%)	88.1	88.4	87.9	88.0	88.0	88.5	88.2	88.0	88.0	87.5	87.5	87.3	87.3	87.3	87.4	87.6	87.3	87.3
CP (g/kg)	19.9	19.9	18.5	18.5	18.5	18.5	18.5	18.5	18.5	18.7	18.7	18.8	18.8	18.9	18.8	18.8	18.8	18.8
Crude fat (g/kg)	5.6	5.6	6.9	7.0	7.1	7.0	7.6	7.1	7.1	4.3	4.3	7.2	7.1	7.2	7.4	8.0	7.2	7.2
Crude ash (g/kg)	5.75	6.07	5.77	5.86	5.89	5.95	6.16	5.94	5.99	5.62	5.87	5.65	5.62	5.63	5.64	5.73	5.65	5.67
Crude fibre (g/kg)	4.06	4.07	3.43	3.52	3.63	3.8	4.44	3.54	3.55	3.31	3.31	3.57	3.65	3.75	392	4.53	3.66	3.67
Starch (g/kg)	334	326	348	342	337	329	291	339	337	374	372	360	357	350	343	310	355	353
Lactose (g/kg)	22.5	22.5	31.9	31.9	31.9	31.9	31.9	31.9	31.9	37.5	37.5	-	-		-	-	-	
Soluble fibres (g/kg)	42.3	45.4	29.8	29.7	29.7	29.8	31.5	30.0	30.1	38.6	38.6	32.5	32.1	32.2	32.2	32.9	32.3	32.5
Insoluble fibers (g/kg)	103.7	106.3	105.8	107.7	109.8	113.7	128.6	109.1	110.0	109.4	109.2	115.0	116.5	118.7	122.0	134.6	117.7	118.6
ME MJ/kg	13.9	13.4	14.2	14.0	14.1	13.5	13.9	14.1	14.2	13.2	13.5	13.6	13.7	13.5	13.5	13.3	13.6	13.6
NE MJ/kg	10.5	10.1	10.7	10.6	10.6	10.0	10.4	10.6	10.8	9.9	10.1	10.2	10.3	10.2	10.2	10.0	10.3	10.2
DE MJ/kg	14.5	14.0	14.8	14.7	14.7	14.2	14.5	14.7	14.8	13.8	14.1	14.2	14.3	14.1	14.1	13.9	14.2	14.2
Composition (g/kg unless otherwise stated)																		
CP	171	171	159	159	159	159	158	158	158	160	160	162	162	164	162	161	162	162
Lys	16.5	16.5	15.4	15.4	15.4	15.3	15.5	15.3	15.3	14.2	14.2	14.2	14.2	14.2	14.2	14.2	14.3	14.3
Met	5.56	5.56	5.31	5.34	5.27	5.32	5.28	5.34	5.34	5.49	5.49	4.42	4.35	4.4	4.32	4.31	4.36	4.36
Met + Cyst	8.58	8.57	8.5	8.59	8.58	8.71	9.01	8.58	8.58	8.67	8.67	7.66	7.66	7.78	7.78	8.07	7.66	7.66

Thr	10.35	10.37		9.76	9.69	9.72	9.76	9.95	9.7	9.71	9.4	9.31	8.72	8.75	8.74	8.71	8.8	8.67	8.69
Try	3.56	3.56		3.61	3.6	3.58	3.56	3.62	3.6	3.6	2.51	2.51	2.93	2.92	2.92	2.88	2.91	2.92	2.93
Val	9.74	9.76		8.88	8.87	8.87	8.86	8.8	8.88	8.89	8.95	8.95	8.67	8.66	8.73	8.65	8.6	8.68	8.7
Macrominerals (g/kg)																			
Calcium	6.24	6.32		7.35	7.41	7.47	7.56	7.69	7.54	7.62	6.61	6.61	8.00	7.81	7.82	7.80	7.82	7.81	7.78
Phosphorus	6.20	6.21		6.60	6.60	6.60	6.60	6.61	6.61	6.60	6.04	6.03	6.40	6.41	6.33	6.34	6.33	6.33	6.32
Na	2.19	2.19		2.07	2.30	2.29	2.31	2.29	2.29	2.29	1.61	1.61	2.30	2.30	2.29	2.31	2.32	2.29	2.29
Mg	1.21	1.23		1.30	1.35	1.39	1.46	1.71	1.35	1.35	1.25	1.25	1.42	1.46	1.52	1.57	1.80	1.45	1.47
Cl	4.39	4.36		5.19	5.54	5.52	5.51	5.52	5.21	5.02	3.79	3.79	5.03	5.01	4.97	5.00	4.96	4.72	4.51
K	8.02	8.03		7.54	7.56	7.58	7.63	8.08	7.65	7.71	8.00	8.01	7.73	7.73	7.81	7.79	8.01	7.82	7.89
Medicinal ZnO (mg/kg)	110	2110		110	110	110	110	110	110	110	110	2110	110	110	110	110	110	110	110
Micromineral premixes (mg/kg)	1170	1170	0	1170	1170	1170	1170	1170	1170	1170	395	395	395	395	395	395	395	395	395

Fermented rapeseed meal (EP100) = FRM; *Ascphyllum nodossum* = AN; negative control = NC; positive control (2500 ppm or 2.5 g/kg or 0.25% ZnO) = PC; [1]AN was included on top of 10% FRM at 0.6% or 1.0% giving 10% FRM +0.6% AN or 10% FRM + 1.0% AN overall supplements; The ingredients 2.5 % mixed cakes, 2.5% sugar beet pulp, 2.50% chicory, 1.0% dry apple, 2.50% Nuklospray® E50 provided in pre-starter and starter diets for NC and PC; 0.25% Arbocel® and 0.10% Globigen® Jump Start in pre-starter for NC; 0.5% microbial Protein, 0.03% Xylanase, 0.03% Ronozyme® VP, 0.1% Mycofix® Plus, 0.5% Tetracid® Dry acid, 0.1% ProPen H, 0.2 % Acidomatrix™ and 12000 IU beta-xylanase provided in starter diets for NC and PC.

Table S2. Effects of pharmacological dose of ZnO, increasing doses of FRM and combination of 10% FRM with increasing doses of AN on growth performance in piglets weaned at 28 days of age.

Performances	Controls		FRM (%)					AN[1](%)	
	NC	PC	8	10	12	15	25	0.6	1.0
Days 42 – 64									
BW at 64, kg	14.8	14.41	17.36	16.13	15.95	16.08	16.55	15.84	16.48
ADG, g/day	267	255	342	353	313	336	329	303	326
Days 65– 92									
BW at d 92, kg	33.55	33.77	34.73	35.43	32.38	35	34.47	31.74	34.1
ADG, g/day	694	717	643	715	609	701	664	589	653

Fermented rapeseed meal (EP100) = FRM; *Ascphyllum nodossum* = AN; negative control = NC; positive control (2500 ppm or 2.5 g/kg or 0.25% ZnO) = PC; [1]AN was included on top of 10% FRM at 0.6% or 1.0% giving 10% FRM +0.6% AN or 10% FRM + 1.0% AN overall supplements.

Table S3. Effects of pharmacological dose of ZnO, increasing doses of FRM and combination of 10% FRM with increasing doses of A.nodossum on number of cases of post-weaning diarrhea and percentage of pigglets exited the weaner units.

Parameters	Treatments								
	Controls		FRM					AN[1] (%)	
	NC	PC	8	10	12	15	25	0.6	1.0
Number of diarrhea cases									
28-42 d of age	6	1	0	4	0	2	2	2	0
143-91 d of age	6	2	12	15	6	10	0	11	22
Duration per cases	1.41	1	1.83	1.55	1.33	2.08	1.33	1.69	1.77
Total no. of diarrhea days	17	3	22	31	8	25	8	22	39
Percentage completed the experiment	77	91	93	86	89	81	89	90	83

Fermented rapeseed meal (EP100) = FRM; *Ascphyllum nodossum* = AN; negative control = NC; positive control (2500 ppm or 2.5 g/kg or 0.25% ZnO) = PC; [1]AN was included on top of 10% FRM at 0.6% or 1.0% giving 10% FRM +0.6% AN or 10% FRM + 1.0% AN overall supplements.

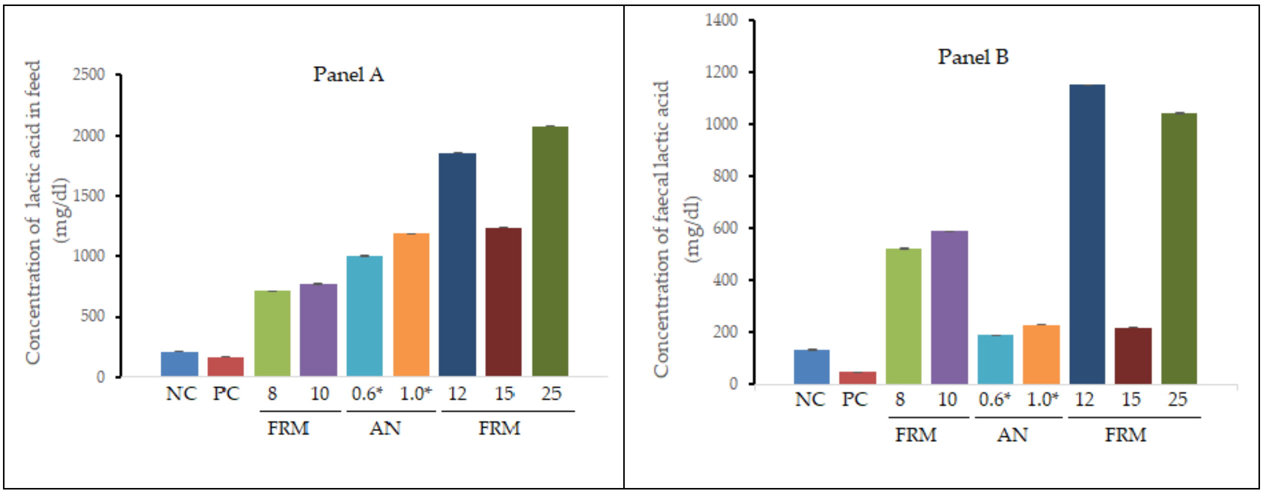

Figure S1. Effect pharmacological doses of ZnO, increasing doses of FRM or combination of 10% FRM with AN on dietary (Panel A) and faecal (PaneL B) lactic acid concentrations; FRM = prefermented rapeseed meal with LAB of 8, 10, 12, 15 and 25%; *Ascphyllum nodossum* = AN; negative control = NC; positive control (2500 ppm or 2.5 g/kg or 0.25% ZnO) = PC; *AN was included on top of 10% FRM at 0.6% or 1.0% giving 10% FRM +0.6% AN or 10% FRM + 1.0% AN overall supplements.

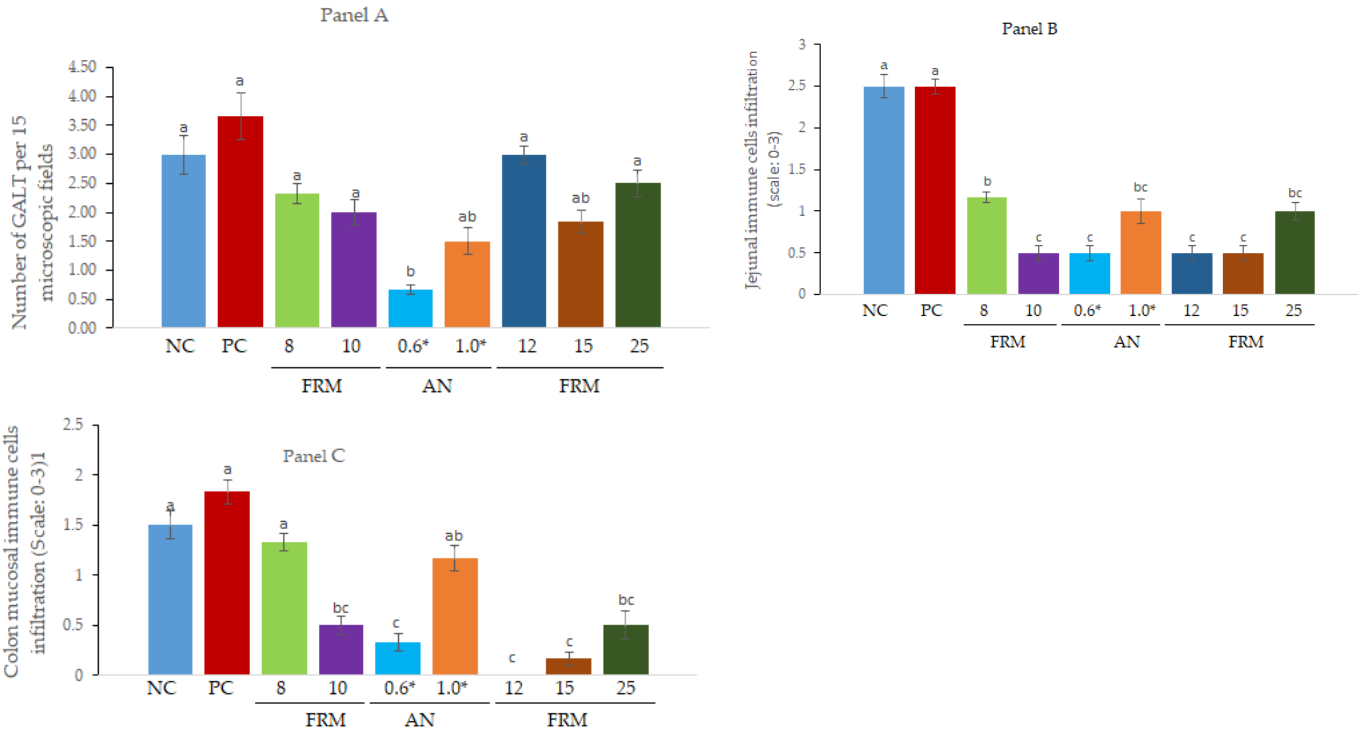

Figure S2. Effects of various doses of FRM with or without AN on density of GALT (Panel A), and jejunal (Panel B) and colonic (Panel C) immune cell infiltrations in piglets (LSM ± SEM) slaughtered two weeks post-weaning; [1,2]Description of scores for jejunal and colonic immune cell infiltrations (0-3) as assessed at x 20: 0 – normal; 1 – Slight infiltration; 2 – Moderate infiltration; 3 – Strong infiltration; FRM = Fermented rapeseed meal inclusion in weaner diets at 8, 10, 12, 15 and 25% of DM; AN= *Ascphyllum nodossum*; NC = negative control; PC = positive control (2500 ppm or 2.5 g/kg or 0.25% ZnO); GALT = gut associated lymphoid tssues; *AN was included on top of 10% FRM at 0.6% or 1.0% giving 10% FRM +0.6% AN or 10% FRM + 1.0% AN overall supplements.
